# Beyond the symptoms: Helping without Medicalizing

**DOI:** 10.1111/aogs.70351

**Published:** 2026-08-28

**Authors:** Sebastian Gidlöf

**Affiliations:** ^1^ Department of Gynecology Ersta Hospital Stockholm Sweden; ^2^ Department of Clinical Science, Intervention and Technology ‐ CLINTEC Karolinska Institutet Stockholm Sweden

Medicine is built on recognizing disease. Yet one of the most important and perhaps most overlooked skills in obstetrics and gynecology is to recognize what is *not* a sign of disease.

Almost every consultation starts with a symptom, but it is not always the symptom that brings the patient to our clinic.

A pregnant woman with nausea may really be concerned whether her baby is well. Perhaps she is not at all interested in symptom relief as long as she knows her symptoms are not caused by any dangers to herself or her baby.

A young woman with irregular bleeding may be concerned that she may ever become pregnant. She may not be interested in regulating her periods if she is reassured that there are no signs of future infertility.

A woman in her midlife may be seeking an explanation of the changes in her body mean, rather than simply only relief from hot flushes.

Obstetrics and gynecology differ from many other medical specialties in that much of what we encounter reflects normal physiology rather than disease, including puberty, menstruation, pregnancy, and menopause. All of these give rise to symptoms that may vary from minor to debilitating, yet they remain part of healthy human biology.

Recognizing physiology from disease must never mean dismissing suffering. Symptoms arising from normal physiological processes may have a severe impact on quality of life and deserve careful attention, thoughtful management, and, when needed, effective treatment. At the same time, physiology deserves to be recognized as just physiology.

Modern obstetrics and gynecology has revolutionized women's health by extraordinary scientific breakthroughs. When we meet suffering, our basic instinct is to act. We prescribe medication, arrange investigations, and plan follow‐up visits. Often, these interventions are exactly what our patients need. But every clinical decision has at least two consequences: the intervention itself and the *meaning it conveys*.

Every prescription, every investigation and every follow‐up visit communicate something about how we, as doctors, understand the patient's body. Mostly the message is appropriate. But sometimes we may, in our honest will to help, unintentionally reinforce the belief that a normal physiological process is a sign of disease. Our interventions therefore shape not only treatment but also the way patients understand their bodies.^1^

I recognize this as I have often done so myself. Both earlier in my career, and sometimes still, I have felt that a consultation is not complete if it does not include a prescription, ultrasound, or a blood test. Merely explaining physiology somehow feels like offering less than modern medicine at its best.

Gradually, experience has taught me otherwise. Explanation is not the absence of action. It is a clinical intervention in itself. When built upon thorough clinical assessment and a thoughtful exclusion of significant pathology, explanation can replace uncertainty with understanding. Explanation may not remove symptoms, but it may often *change how they are experienced*.^1^, ^2^

A normal examination answers only the question it was intended for, but it does not necessarily answer the question the patient came to ask. Perhaps it is here that clinical judgment is most evident. It is not only about recognizing disease but also the ability to recognize health—to distinguish pathology from physiology and decide which intervention is most likely to help in both the short term and in the long run. Sometimes that intervention may be medication, surgery, or another investigation. Sometimes it may be knowledge.

Person‐centered care does not begin with deciding which treatment to offer, but by understanding what the patient hopes the consultation will achieve. Only then can the doctor, together with the patient, decide whether the best way forward is treatment, investigation, watchful waiting, or an explanation.^2^ Protecting patients from unnecessary medicalization while remaining vigilant for significant disease is an essential part of good clinical practice.^3^

Modern medicine has become extraordinarily good at detecting abnormalities and it is a progress worth celebrating. Yet, increasingly sensitive diagnostic methods inevitably blur the boundary between normal variation and disease. This opens the door for overdiagnosis and interventions that may offer little benefit while changing how healthy people perceive themselves.^4^

Many patients have a strong belief that a doctor would only treat something that needs treatment and investigate symptoms further if they represent true signs of disease. We must therefore keep in mind that when we prescribe, arrange investigations, perform further examinations, we may unintentionally do harm by reinforcing the patient's belief that the symptom is caused by pathology rather than physiology.

As obstetricians and gynecologists, we accompany women through many of life's most important biological transitions. Our responsibility, therefore, extends beyond diagnosing disease. It also includes recognizing health, strengthening confidence in the body's remarkable ability to adapt, and helping women to distinguish danger from the sometimes uncomfortable realities of normal physiology. Sometimes the most important thing we can offer is the recognition of normal physiology and health.

## Data Availability

The data that support the findings of this study are available from the corresponding author upon reasonable request.
